# Cardiovascular risk management in rheumatoid and psoriatic arthritis: online survey results from a national cohort study

**DOI:** 10.1186/s41927-018-0032-9

**Published:** 2018-09-06

**Authors:** Premarani Sinnathurai, Alexandra Capon, Rachelle Buchbinder, Vibhasha Chand, Lyndall Henderson, Marissa Lassere, Lyn March

**Affiliations:** 1 Institute of Bone and Joint Research, Kolling Institute, Northern Sydney Local Health District, Sydney, NSW Australia; 2 Department of Rheumatology, Royal North Shore Hospital, Reserve Road, St Leonards, NSW 2065 Australia; 3 Sydney Medical School, University of Sydney, Sydney, NSW Australia; 4 Monash Department of Clinical Epidemiology, Cabrini Institute, Malvern, VIC Australia; 5 Department of Epidemiology and Preventive Medicine, School of Public Health and Preventive Medicine, Monash University, Clayton, VIC Australia; 6 Centre of Cardiovascular Research & Education in Therapeutics, School of Public Health and Preventive Medicine, Monash University, Clayton, VIC Australia; 7 School of Public Health and Community Medicine, University of New South Wales, Sydney, NSW Australia; 8 Department of Rheumatology, St George Hospital, Kogarah, NSW Australia

**Keywords:** Rheumatoid arthritis, Psoriatic arthritis, Cardiovascular diseases, Medications, Diet, Physical activity

## Abstract

**Background:**

Chronic inflammatory arthritis is associated with increased cardiovascular (CV) morbidity and mortality. Pharmacological management and healthy lifestyle modification is recommended to manage these risks, but it is not known how often these are utilised and whether there is any difference in their use between patients with different types of arthritis. The aim of this study was to determine and compare the proportion of participants with rheumatoid arthritis (RA) and psoriatic arthritis (PsA) receiving pharmacological or lifestyle management strategies for CV risk factors. The secondary objective was to identify factors associated with use of management strategies.

**Methods:**

A survey was sent to online participants in the Australian Rheumatology Association Database, a voluntary national registry for inflammatory arthritis. Participants were asked whether they took medications for hypertension, hyperlipidaemia and diabetes, and to report their height, weight, level of physical activity, and dietary changes made. The use of management strategies was compared between participants with RA and PsA. Logistic regression analyses were performed to identify factors associated with physical activity and dietary changes.

**Results:**

There were 858 respondents with RA and 161 with PsA (response rate 64.5%). Pharmacological treatment was reported by 93% of participants with hypertension and 70% with hyperlipidaemia. All participants with diabetes reported being managed with dietary modification, pharmacological treatment, or a combination of both. Adequate physical activity was reported by 50.8%. Only 27% of overweight or obese participants reported making any dietary change for their health in the past year. There was no difference between RA and PsA in reported utilisation of management strategies. Hyperlipidaemia and being overweight were associated with making dietary change. Obesity and arthritis disease activity were negatively associated with physical activity.

**Conclusions:**

Most participants with RA and PsA reported using pharmacological treatment for CV risk factors. Relatively few reported using lifestyle modifications. Targeted lifestyle interventions should be implemented for RA and PsA patients.

**Electronic supplementary material:**

The online version of this article (10.1186/s41927-018-0032-9) contains supplementary material, which is available to authorized users.

## Background

Chronic inflammatory arthritis is associated with increased cardiovascular (CV) morbidity and mortality [1]. Systemic inflammation may cause insulin resistance and endothelial dysfunction, which then leads to atherosclerosis and vascular disease [2]. An increased risk of CV mortality is well established in rheumatoid arthritis (RA). A meta-analysis of observational studies, published in 2008, demonstrated 50% increased risk of CV death in patients with RA (meta-standardised mortality ratio (SMR) 1.50, 95% confidence interval (CI) 1.39–1.61) compared with the general population [3]. Both traditional CV risk factors and markers of severity of RA are predictors of future CV events [4]. In recent years, there seems to have been a changing trend. In two cohorts in North America, mortality from CV disease in patients with incident RA since the year 2000 appears to be similar to that in general population controls [5, 6]. It is not known whether this trend may be attributed to improved management of RA or more stringent screening and treatment for CV risk factors.

Psoriatic arthritis (PsA) is associated with an increased risk of the metabolic syndrome and other CV risk factors [7–10]. Previous analysis from the Australian Rheumatology Association Database (ARAD) showed that in this cohort, diabetes mellitus and high cholesterol were more common in participants with PsA than RA [11]. In the Consortium of Rheumatology Researchers of North America (CORRONA) Registry, PsA was associated with higher rates of obesity, diabetes mellitus and hypertriglyceridaemia, compared with RA [12]. Due to this high prevalence of traditional CV risk factors in PsA, it might be expected that CV mortality in PsA might be increased at a rate similar to, or even higher than in RA. However, the evidence relating to mortality in PsA is mixed with SMRs ranging from 0.82–1.62 [13–15]. Several studies found an increase in all-cause mortality, with CV disease the most common cause of death [16, 17]. One longitudinal cohort study demonstrated an overall increase in mortality, with a trend for improvement in survival over time [18]. However, other studies have found no increase in mortality compared with the general population [19, 20].

The reasons for this discrepancy in reported mortality in PsA, and for the apparent difference in mortality between PsA and RA are not yet known. There may be inherent differences in pathophysiology contributing to the difference observed between RA and PsA, although both are associated with chronic systemic inflammation. PsA is a heterogeneous disease and variable disease phenotype may contribute to the differences in mortality. Alternatively, variability in the management of CV risk factors may account for some of the diversity in mortality trends.

A EULAR task force produced recommendations for the management of CV risk in patients with inflammatory arthritis, which were updated in 2016 [21, 22]. The evidence available from their systematic literature review was far greater for RA than for PsA or ankylosing spondylitis. They advise CV risk assessment and management should be performed in accordance with national guidelines. They advise that healthy diet, regular exercise and smoking cessation should be recommended, based on accumulating evidence that physical inactivity is common in patients with RA and exercise may have beneficial effects on CV disease and systemic inflammation. The Group for Research and Assessment of Psoriasis and Psoriatic Arthritis (GRAPPA) recommend that all PsA patients should be encouraged to achieve and maintain a healthy body weight [23]. Similarly, recommendations from Spanish expert panels emphasise the importance of screening for CV disease and management in a multidisciplinary environment including promotion of regular exercise, healthy body weight and smoking cessation for patients with RA and PsA [24–26]. The Australian guidelines for the management of absolute CV disease risk also include a consensus-based recommendation that lifestyle advice and support be given to all adults, even those assessed to have low CV risk [27].

It is not known how often pharmacological management strategies and/or lifestyle modifications are currently being used by patients with inflammatory arthritis to manage CV risk, and whether there is any difference in the utilisation of these strategies in patients with RA or other inflammatory arthritides such as PsA. The primary objective of this study was to describe and compare the proportion of participants in ARAD with RA or PsA utilising pharmacological and/or lifestyle management strategies for CV risk factors. The secondary objective was to explore factors that are associated with reported utilisation of lifestyle modifications which can reduce CV risk.

## Methods

ARAD is a voluntary national registry which collects longitudinal health information from people with inflammatory arthritis, including PsA, RA, ankylosing spondylitis and juvenile idiopathic arthritis, with diagnosis being confirmed by the treating rheumatologist [28]. The database was established in 2003 and has been previously described in detail [28]. Briefly, participants complete questionnaires every 6–12 months in online or paper format. These questionnaires include demographic data, past medical history, treatment for arthritis, adverse effects, infections and malignancies. Patient-reported pain is collected using pain visual analogue scale (VAS, 0 = no pain to 100 = pain as bad as it could be). Self-reported disease activity is also collected using global assessment VAS (0 = none to 100 = extreme). Written consent is obtained from all participants. Rigorous quality control and validation processes are undertaken to check and follow up any missing data to ensure database quality.

The Heart Health Survey was sent to all online ARAD participants with RA (*n* = 1295) and PsA (*n* = 285). This cross-sectional survey was sent in September 2015, with a reminder sent one month later to non-respondents. The survey was closed in December 2015. The survey asked participants about whether they took any medications for selected cardiovascular risk factors, and also about dietary changes and level of physical activity. Participants were asked:

- if they took any medications for hypertension, hyperlipidaemia and diabetes (yes or no);
- whether they had made any dietary changes for their health in the last year, such as seeing a dietician or participating in a weight loss program (yes or no); if they had made a dietary change, participants were asked if they had attended a weight loss program run by a dietician or a commercial program, whether they had used meal replacements, had bariatric surgery, or attended an exercise program;
- how often they performed moderate physical activity, defined as physical activity associated with a moderate, noticeable increase in the depth and rate of breathing while still being able to whistle or talk comfortably. They were given options ranging from no physical activity to 30 min of moderate physical activity every day;
- if there were any medical conditions which limited their ability to participate in physical activity, including heart conditions, breathing difficulty, problems relating to a previous stroke, their arthritis, other conditions, or if there were no medical conditions limiting their activity;
- self-reported weight and height.

For all survey recipients, demographic information, arthritis medications, comorbidities and self-reported global assessment of disease activity (reported on a 0 to 100 VAS where a higher score indicates more disease activity) and pain (0 to 100 VAS where a higher score indicates more pain) were extracted from their most recent ARAD entry.

Statistical analysis was performed using IBM SPSS Statistics 22. Descriptive analyses were used to determine the proportion of participants with CV risk factors including smoking, hypertension, hyperlipidaemia, and diabetes. Self-reported height and weight were used to calculate body mass index (BMI) for respondents. Participants were classified as overweight if they had a BMI greater than or equal to 25 kg/m^2^, and obese if their BMI was greater than or equal to 30 kg/m^2^ [29]. Adequate physical activity was defined as performing 30 min of moderate intensity physical activity at least three days of the week. This definition was based on a consensus recommendation developed for people with arthritis [30], and is less stringent than the World Health Organisation guideline for adults which is a minimum of 150 min of moderate intensity physical activity per week [31]. For between group comparisons, Chi-squared and student T-tests were used for categorical and continuous variables respectively. *P* values of 0.05 or less were considered statistically significant.

Logistic regression was used to identify factors which were associated with physical activity and dietary changes. For these regression analyses, participants with RA and PsA were combined. However, only participants who had completed an ARAD questionnaire within 30 days of the Heart Health Survey, and therefore had recent measures of self-reported global assessment of disease activity and pain, were included in the regression analyses. Univariate analysis was first performed on potential predictors including age, gender, education level and employment status, diagnosis (RA or PsA), arthritis treatments (methotrexate, prednisone, biologic disease modifying anti-rheumatic drug (DMARD)), CV risk factors (hypertension, hyperlipidaemia, diabetes, smoking, obesity), disease duration, and disease activity as measured by patient global assessment and pain VAS. Low disease activity was defined as a patient global assessment score less than or equal to 20. Variables which were associated with the outcome of interest with *p* ≤ 0.25 in the univariate logistic regression were entered into a multivariate logistic regression model and non-significant covariates removed via backwards stepwise elimination until only significant variables (*p* < 0.05) remained in the final model [32].

## Results

**Fig. 1:**
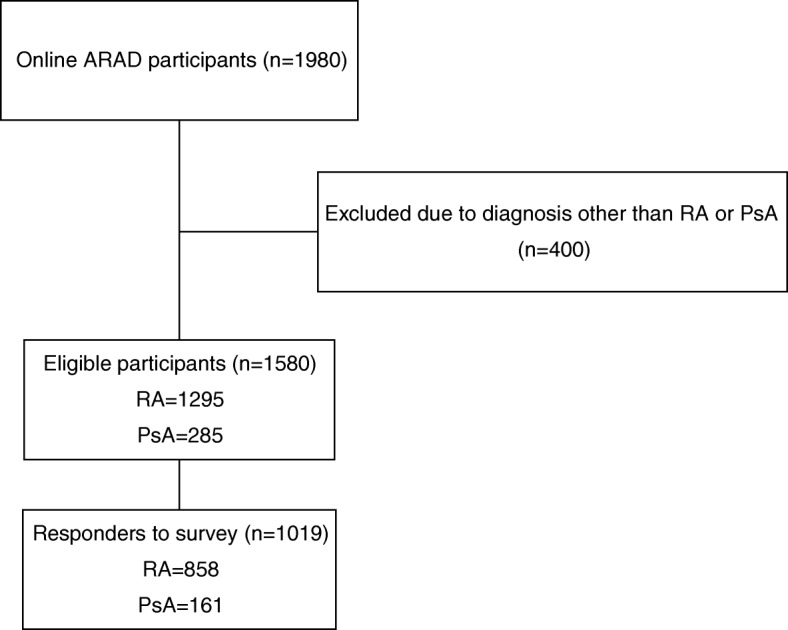
Flow diagram of participant inclusion from ARAD. ARAD: Australian Rheumatology Association Database, RA: rheumatoid arthritis, PsA: psoriatic arthritis

Overall 1019 participants responded to the survey (overall response rate 64.5%), including 858 with RA (response rate 66.3%) and 161 with PsA (response rate 56.5%) (Fig. 1). Table 1 summarises demographic information for responders and non-responders. Overall, responders were older, and had longer disease duration. Responders were more likely to be taking a biologic DMARD and methotrexate, but less likely to be smoking or working or studying full time.

**Table 1:** Characteristics of Responders and Non-Responders to Heart Health Survey

	Responders (*N* = 1019)	Non-Responders (*N* = 561)	*p*
	Mean (SD)	Mean (SD)	
Age, years	58 (11)	53 (13)	< 0.001
Disease duration, years	18 (11)	16 (10)	0.001
	N (%)	N (%)	
Diagnosis			0.002
RA	858 (84.2)	437 (77.9)	
PsA	161 (15.8)	124 (22.1)	
Female	752 (73.8)	407 (72.5)	0.6
University/tertiary education	605 (59.4)	313 (55.8)	0.17
Full time work or study	281 (27.6)	214 (38.1)	< 0.001
Current biologic DMARD	752 (73.8)	380 (67.7)	0.01
Current methotrexate	662 (65.0)	334 (59.5)	0.03
Current prednis(ol)one	311 (30.5)	188 (33.5)	0.22
Current smoker	62 (6.1)	49 (8.7)	0.05
Hypertension	332 (32.6)	160 (28.5)	0.10
Diabetes	87 (8.5)	42 (7.5)	0.5
Hyperlipidaemia	183 (18.0)	85 (15.2)	0.16

*SD* standard deviation, *RA* rheumatoid arthritis, *PsA* psoriatic arthritis, *DMARD* disease modifying antirheumatic drug

Characteristics of responders, stratified by diagnosis, are shown in Table 2. RA responders were slightly older than PsA responders, and had slightly longer disease duration. There were more female respondents with RA, in keeping with the known epidemiology of this disease. Participants with PsA were more likely than those with RA to be overweight or obese (131/161, 81.4% vs 564/858, 66.0%, *p* < 0.001). Approximately half of all respondents (518/1019, 50.8%) were classified as physically active and the proportion of physically active respondents was similar in both disease groups. However, 189/1019 (18.5%) reported that they had not performed any moderate intensity physical activity in the last week. The prevalence of other CV risk factors was similar between the two groups. Current cigarette smoking was reported by 6.1% of participants. Data regarding whether participants had received smoking cessation advice or management strategies were not collected in this study.

**Table 2:** Demographics and cardiovascular risk factors for RA and PsA Responders

	RA (*N* = 858)	PsA (*N* = 161)	*p*
	Mean (SD)	Mean (SD)	
Age, years	58 (12)	55 (10)	< 0.001
Disease duration, years	18 (11)	15 (9)	0.005
	N (%)	N (%)	
Female	657 (76.6)	95 (59.0)	< 0.001
University/tertiary education	504 (58.7)	101 (62.7)	0.3
Full time work or study	214 (24.9)	67 (41.6)	< 0.001
Current biologic DMARD	627 (73.1)	125 (77.6)	0.23
Current methotrexate	576 (67.1)	86 (53.4)	0.001
Current prednis(ol)one	295 (34.4)	16 (9.9)	< 0.001
Current smoker	52 (6.1)	10 (6.2)	0.9
Hypertension	279 (32.5)	53 (32.9)	0.9
Diabetes	73 (8.5)	14 (8.7)	0.9
Hyperlipidaemia	157 (18.3)	26 (16.1)	0.5
Overweight or obese	564 (66.0)	131 (81.4)	< 0.001
Physically active	418 (48.7)	83 (51.6)	0.5

*RA* rheumatoid arthritis, *PsA* psoriatic arthritis, *SD* standard deviation, *DMARD* disease modifying antirheumatic drug

Pharmacological treatments, dietary changes and physical activity reported by participants are shown in Table 3. Most participants with hypertension or hyperlipidaemia reported taking medications for these risk factors (93% and 70% respectively). All participants who reported having diabetes reported being managed with dietary modification, pharmacological treatment, or a combination of the two. Only about a quarter of RA and PsA participants who were overweight or obese reported that they had made any dietary change for their health in the past year. For those who had made a change, use of meal replacements was the most commonly reported strategy, and 57/151 (37.8%) RA and 11/36 (30.6%) PsA reported participating in an exercise program.

**Table 3:** Reported treatment for cardiovascular risk factors

CV risk factor	Treatment	Responders with reported risk factor reporting receiving treatment		*p*
		RA	PsA	
		N (%)	N (%)	
Hypertension	Pharmacological	259/279 (92.8)	50/53 (94.3)	0.7
Diabetes	Diet and/or pharmacological	73/73 (100.0)	14/14 (100.0)	N/A
Hyperlipidaemia	Pharmacological	109/157 (69.4)	19/26 (73.1)	0.7
Overweight or obese	Any dietary change in the last year	151/564 (26.8)	36/131 (27.5)	0.9
	Commercial weight loss program	21/564 (3.7)	3/131 (2.3)	0.4
	Health professional weight loss program	44/564 (7.8)	7/131 (5.3)	0.3
	Meal replacements	53/564 (9.4)	10/131 (7.6)	0.5
	Exercise program	57/564 (10.1)	11/131 (8.4)	0.6
	Bariatric surgery	4/564 (0.7)	1/131 (0.8)	0.9

Treatment for hypertension, diabetes, hyperlipidaemia and being overweight or obese in RA and PsA respondents to the Heart Survey and a comparison of the two disease groups

*CV* cardiovascular, *RA* rheumatoid arthritis, *PsA* psoriatic arthritis, *N/A* not applicable

In all participants, arthritis was by far the most commonly reported factor limiting physical activity (703/1019, 69.0%). In those who were classified as performing insufficient physical activity, 361/501 (72.1%) reported that their arthritis limited their ability to participate in physical activity. Only 26 (2.6%) reported heart conditions limited their activity, 14 (1.4%) reported stroke as a limiting factor and 95 (9.3%) reported breathing difficulties. Out of all respondents, 255 (25.0%) reported there were no medical conditions limiting their physical activity, including 100/501 (20.0%) who had insufficient physical activity. There were no significant differences in the utilisation of pharmacological or lifestyle management strategies between the RA and PsA groups.

The results for univariate and multivariate logistic regression on physical activity are shown in Table 4. There were 275 participants who had completed an ARAD questionnaire within 30 days of the Heart Health Survey and could therefore be included in the logistic regression. There were no significant demographic differences between the participants included in the logistic regression and those who were excluded (data not shown). In univariate analysis, lower self-reported disease activity was associated with higher odds of being physically active. Hypertension and being overweight or obese were associated with lower odds of physical activity. Self-reported pain and global assessment of disease activity were closely correlated (Pearson Correlation coefficient = 0.86) and therefore these variables were entered into separate multivariate regression models. However, in the multivariate analysis, pain was not significantly associated with physical activity, and therefore the results of the final model including self-reported global assessment of disease activity are presented in Table 4. In the final model, there was a statistically significant inverse association between being physically active and being overweight or obese, while low self-reported disease activity was significantly positively associated with being physically active.

**Table 4:** Odds and adjusted odds ratios for demographic and clinical characteristics associated with being physically active

Univariate analysis			Multivariate analysis (baseline model)*		Multivariate analysis (final model)*	
Variable	OR (95% CI)	*p*	Adjusted OR (95% CI)	*p*	Adjusted OR (95% CI)	*p*
Sex (female vs male)	1.09 (0.70–2.05)	0.5	–	–	–	–
Diagnosis (PsA vs RA)	0.80 (0.42–1.52)	0.5	–	–	–	–
Current biologic DMARD	0.99 (0.58–1.69)	1.0	–	–	–	–
Current methotrexate	1.31 (0.81–2.12)	0.3	–	–	–	–
Current prednis(ol)one	0.90 (0.54–1.48)	0.7	–	–	–	–
Current hypertension	0.58 (0.35–0.98)	0.04	0.65 (0.38–1.11)	0.11	–	–
Current hyperlipidaemia	1.20 (0.67–2.14)	0.6	–	–	–	–
Current diabetes	1.04 (0.44–2.49)	0.9	–	–	–	–
Current smoker	1.22 (0.40–3.73)	0.7	–	–	–	–
Overweight or obese	0.51 (0.31–0.84)	0.01	0.59 (0.35–0.99)	0.04	0.56 (0.33–0.93)	0.03
University/tertiary education	1.20 (0.74–1.93)	0.5	–	–	–	–
Full time work or study	0.98 (0.57–1.66)	0.9	–	–	–	–
Age ≥ 65 years	1.17 (0.70–1.94)	0.6	–	–	–	–
Disease duration ≥5 years	0.68 (0.27–1.75)	0.4	–	–	–	–
Low self-reported disease activity (VAS ≤ 20)	1.88 (1.14–3.09)	0.01	1.69 (1.01–2.82)	0.05	1.71 (1.03–2.85)	0.04
Low pain (VAS ≤20)	1.57 (0.95–2.61)	0.08	–	–	–	–

Combined analysis including RA and PsA respondents, *N* = 275

*OR* odds ratio, *CI* confidence interval, *RA* rheumatoid arthritis, *PsA* psoriatic arthritis, *DMARD* disease modifying antirheumatic drug, *VAS* visual analogue scale

^*^Variables with *p* ≤ 0.25 in the univariate logistic regression were entered into the multivariate logistic regression baseline model and non-significant covariates removed via backwards stepwise elimination until only significant covariates remained in the final model. Stepwise backwards elimination process can be seen in Additional file 1. Nagelkerke R^2^ for model = 0.06

Results of the logistic regression on dietary change are shown in Table 5. In univariate analysis, reporting high cholesterol or being overweight or obese were associated with a higher odds of having made a dietary change for health reasons in the last year. However, current biologic DMARD treatment and self-reported low disease activity were associated with lower odds of having made a dietary change. In multivariate analysis, the associations with biologic DMARD treatment, high cholesterol and being overweight or obese persisted, but the association with self-reported low disease activity and low pain were no longer statistically significant.

**Table 5:** Odds and adjusted odds ratios for demographic and clinical characteristics associated with making dietary change

Univariate analysis			Multivariate analysis (baseline model)*		Multivariate analysis (final model)*	
Variable	OR (95% CI)	*p*	Adjusted OR (95% CI)	*p*	Adjusted OR (95% CI)	*p*
Sex (female vs male)	1.72 (0.81–3.62)	0.16	1.90 (0.86–4.20)	0.11	–	–
Diagnosis (PsA vs RA)	0.87 (0.38–1.98)	0.7	–	–	–	–
Current biologic DMARD	0.44 (0.23–0.82)	0.01	0.42 (0.21–0.82)	0.01	0.36 (0.19–0.71)	0.003
Current methotrexate	1.12 (0.61–2.06)	0.7	–	–	–	–
Current prednis(ol)one	0.82 (0.43–1.57)	0.6	–	–	–	–
Current hypertension	1.63 (0.88–3.04)	0.12	1.26 (0.63–2.51)	0.51	–	–
Current hyperlipidaemia	2.08 (1.07–4.06)	0.03	2.06 (0.99–4.31)	0.05	2.20 (1.09–4.44)	0.03
Current diabetes	1.60 (0.60–4.31)	0.4	–	–	–	–
Current smoker	0.73 (0.16–3.42)	0.7	–	–	–	–
Overweight or obese	3.91 (1.76–8.67)	0.001	4.08 (1.76–9.47)	0.001	4.49 (1.97–10.26)	< 0.001
University/tertiary education	0.94 (0.52–1.72)	0.9	–	–	–	–
Full time work or study	0.94 (0.48–1.85)	0.9	–	–	–	–
Age ≥ 65 years	1.10 (0.59–2.08)	0.8	–	–	–	–
Disease duration ≥5 years	1.33 (0.37–4.73)	0.7	–	–	–	–
Self-reported low disease activity (VAS ≤ 20)	0.43 (0.22–0.87)	0.02	0.55 (0.26–1.16)	0.12	–	–
Low pain (VAS ≤20)	0.46 (0.22–0.94)	0.03	–	–	–	–

Combined analysis including RA and PsA respondents, *N* = 275

*OR* odds ratio, *CI* confidence interval, *RA* rheumatoid arthritis, *PsA* psoriatic arthritis, *DMARD* disease modifying antirheumatic drug, *VAS* visual analogue scale

^*^Variables with *p* ≤ 0.25 in the univariate logistic regression were entered into the multivariate logistic regression baseline model and non-significant covariates removed via backwards stepwise elimination until only significant covariates remained in the final model. Stepwise backwards elimination process can be seen in Additional file 1. Nagelkerke R^2^ for model = 0.15

## Discussion

CV risk factors were common in this cohort with RA and PsA, in keeping with other reported RA and PsA cohorts [10, 33]. Most of the participants with hypertension, hyperlipidaemia and diabetes reported receipt of pharmacological treatment, and there was no difference in utilisation rates between RA and PsA. Few studies have examined the management of CV risk factors in inflammatory arthritis. Using data from The Health Improvement Network medical record database in the United Kingdom [34], Jafri et al. reported similarly high utilisation of pharmacotherapy; approximately 85% of patients with hypertension, 65% with hyperlipidaemia and 45% with diabetes received prescriptions for pharmacotherapy. There was no difference in the frequency of prescription of therapy when comparing the PsA, RA and general population control cohorts in that study, and use of lifestyle modification was not examined as this was not readily identifiable in the coded database. In this study from ARAD, the use of lifestyle modifications known to improve CV risk was assessed by participant self-report. Only about half of the respondents reported adequate levels of physical activity, and less than one third of patients who were overweight or obese report having made a dietary change for their health in the last year.

Respondents reporting high cholesterol or obesity were more likely to have made a dietary change than those without these risk factors, and participation in a weight loss program run by a health professional was the most commonly reported method of dietary change. Use of biologic DMARDs was negatively associated with making a dietary change, but the reasons for this association are unclear. The analyses identifying factors associated with physical activity and dietary changes were exploratory, and it is possible that some significant findings may have occurred by chance.

On a global level, physical inactivity has been described as a pandemic which should be a public health priority [35]. However, arthritis has been recognised as a barrier to physical activity in patients with obesity, heart disease and diabetes [36–38]. This study from ARAD highlights the low level of physical activity in patients with inflammatory arthritis, despite the known health benefits [39, 40]. Approximately half of all respondents were classified as physically inactive, and obesity was associated with being physically inactive. Arthritis was the most commonly reported condition limiting physical activity and those with low self-reported disease activity were more likely to be physically active. However, other comorbidities and demographic factors including age and level of education were not significantly associated with physical activity. In a cross-sectional international study of patients with RA published in 2008, even higher rates of physical inactivity were reported than in this Australian cohort; only 13.8% of patients reported physical exercise three or more times weekly [41]. In the 2002 National Health Interview Survey in the United States, 63% of adults with arthritis did not meet the arthritis expert panel recommendation for physical activity, compared with 61% of those without arthritis [42]. Using data from the 2000 Behavioral Risk Factor Surveillance System survey in the general United States population, Hootman et al. reported 30.8% of people with arthritis are completely inactive, compared to 25.8% of those without arthritis [43].

There are some limitations to this study. Due to the self-reported nature of ARAD, clinical information such as blood pressure readings and blood lipid or glucose levels were not available. It is possible that some respondents in ARAD had undiagnosed CV risk factors which were not detected through self-report. Also, it was not possible to assess the severity of CV risk factors, or the adequacy of treatment. In a Dutch cross-sectional cohort study in which blood pressure and cholesterol levels were measured, 42% of patients with RA received inadequate lipid-lowering and/or antihypertensive treatment, based on Dutch CV risk management guidelines [44]. Online ARAD participants who did not respond to the survey were more likely to be working full time and less likely to be taking biologic DMARDs than survey responders. It is therefore possible that this non-responder group had less severe disease and may have different patterns of physical activity or CV risk management. The number of participants was relatively small, particularly in the PsA subgroup, and the number of participants who were included in the regression analysis, which may have affected the analysis. Furthermore, participants in ARAD are predominantly Caucasian, with English as their first language and over half of respondents had a university or other tertiary level education. Therefore, the findings from this study may not be generalisable to the wider population of people with RA and PsA.

The Heart Health survey focussed on medical conditions limiting physical activity but did not investigate social, environmental or psychological barriers to physical activity and dietary change. The R^2^ values for the regression models were low (0.06 for physical activity and 0.15 for dietary change) indicating that there are factors which are not accounted for which may be associated with lifestyle modifications. In other published studies, older age, lower education, self-efficacy and pain have been associated with physical activity status in people with arthritis [42, 45]. A qualitative study among adults with arthritis identified a multitude of physical, psychological, social and environmental barriers to exercise [46]. Pain and lack of exercise programs or facilities specifically for people with arthritis emerged in almost all groups. Patients with RA have reported that fear for safety, and uncertainty regarding what type and amount of activity is recommended is a barrier to participating in physical activity or exercise [47].

There is a need for further research to identify barriers that prevent patients from taking up dietary modification and regular physical activity, so that appropriate, targeted interventions can be designed to combat these problems. The population health strategies encouraging physical activity in the general population are not always appropriate for those with arthritis who face particular challenges relating to their disease. However, even light and very light intensity exercise is associated with favourable cardiovascular markers and lower disease activity in rheumatoid arthritis [48]. Although the current goal in treat to target strategies in RA [49] and PsA [50] is to maximise long-term health-related quality of life, including physical function and participation in work and social activities, the primary method to achieve this goal is focussed on the use of DMARDs to control inflammation. Multidisciplinary models of care are needed to address not only pharmacological treatment for inflammation, but also target achievable physical activity and healthy weight goals to improve patient outcomes, both disease-specific and relating to long-term CV risk.

## Conclusions

In this study, most participants with RA and PsA were managing their CV risk factors using pharmacological treatments. However, relatively few had undertaken lifestyle modifications to improve their CV risk. There was no difference in the utilisation of these management strategies between those with RA and PsA. Treating clinicians should look beyond pharmacological management and address targeted lifestyle interventions for their RA and PsA patients.

## Additional file

Additional file 1: Stepwise regression for multivariate logistic regression models. Stepwise backwards elimination of covariates to build final multivariate logistic regression models for physical activity and dietary change. (PDF 234 kb)

## Abbreviations

ARAD: Australian Rheumatology Association Database
BMI: Body mass index
CI: Confidence interval
CORRONA: Consortium of Rheumatology Researchers of North America
CV: Cardiovascular
DMARD: Disease modifying antirheumatic drug
GRAPPA: Group for Research and Assessment of Psoriasis and Psoriatic Arthritis
OR: Odds ratio
PsA: Psoriatic arthritis
RA: Rheumatoid arthritis
SD: Standard deviation
SMR: Standardised mortality ratio
VAS: Visual analogue scale
