# Supplementary material for: Cardiovascular risk management in rheumatoid and psoriatic arthritis: online survey results from a national cohort study
Source: BMC Rheumatol. 2018 Sep 6;2:25. doi: 10.1186/s41927-018-0032-9 (PMC6390588; doi:10.1186/s41927-018-0032-9)
Supplement: Supplementary file 1 — Stepwise regression for multivariate logistic regression models. Stepwise backwards elimination of covariates to build final multivariate logistic regression models for physical activity and dietary change. (PDF 234 kb) [file 41927_2018_32_MOESM1_ESM.pdf]

Multivariate model on physical activity: stepwise backwards elimination to build the final model.

Step 1: all variables with p≤0.25 on univariate analysis (using global VAS, not pain VAS as these were too closely correlated to be included in the same model).

Variable	Adjusted OR (95% CI)	p
Overweight or obese	0.59 (0.35-0.99)	0.04
Self-reported low disease activity (VAS<-20)	1.69 (1.01-2.82)	0.05
Current hypertension	0.65 (0.38-1.11)	0.11

Step 2: removal of hypertension, as not significant predictor

Variable	Adjusted OR (95% CI)	p
Overweight or obese	0.56 (0.33-0.93)	0.03
Self-reported low disease activity (VAS<-20)	1.71 (1.03-2.85)	0.04

All remaining covariates are now statistically significant and this is the final model, as shown in Table 4.

Multivariate model on dietary change: stepwise backwards elimination to build the final model.

Step 1: all variables with p≤0.25 on univariate analysis (using global VAS, not pain VAS as these were too closely correlated to be included in the same model).

Variable	Adjusted OR (95% CI)	p
Overweight or obese	4.08 (1.76-9.47)	0.001
Self-reported low disease activity (VAS<-20)	0.55 (0.26-1.16)	0.12
Sex (female vs male)	1.90 (0.86-4.20)	0.11
Current biologic DMARD	0.42 (0.21-0.82)	0.01
Current hypertension	1.26 (0.63-2.51)	0.51
Current hyperlipidaemia	2.06 (0.99-4.31)	0.05

Step 2: removal of hypertension, as the least significant variable

Variable	Adjusted OR (95% CI)	p
Overweight or obese	4.18 (1.81-9.66)	0.001
Self-reported low disease activity (VAS<-20)	0.54 (0.26-1.13)	0.11
Sex (female vs male)	1.88 (0.85-4.13)	0.12
Current biologic DMARD	0.42 (0.21-0.83)	0.03
Current hyperlipidaemia	2.20 (1.08-4.48)	0.03

Step 3: removal of sex as the least significant variable

Variable	Adjusted OR (95% CI)	p
Overweight or obese	4.02 (1.74-9.26)	0.001
Self-reported low disease activity (VAS<-20)	0.57 (0.27-1.19)	0.13
Current biologic DMARD	0.39 (0.20-0.75)	0.01
Current hyperlipidaemia	2.20 (1.08-4.46)	0.03

Step 4: removal of self-reported low disease activity as the least significant variable

Variable	Adjusted OR (95% CI)	p
Overweight or obese	4.49 (1.97-10.26)	<0.001
Current biologic DMARD	0.36 (0.19-0.71)	0.003
Current hyperlipidaemia	2.20 (1.09-4.44)	0.03

All remaining covariates are now statistically significant and this is the final model, as presented in Table 5.
